# Functional oropharyngeal sensory disruption interferes with the cortical control of swallowing

**DOI:** 10.1186/1471-2202-8-62

**Published:** 2007-08-02

**Authors:** Inga K Teismann, Olaf Steinstraeter, Kati Stoeckigt, Sonja Suntrup, Andreas Wollbrink, Christo Pantev, Rainer Dziewas

**Affiliations:** 1 Institute for Biomagnetism and Biosignalanalysis, University of Muenster, Malmedyweg 15, 48149 Muenster, Germany; 2 Department of Neurology, University of Muenster, Albert-Schweitzer-Str. 33, 48149 Munster, Germany

## Abstract

**Background:**

Sensory input is crucial to the initiation and modulation of swallowing. From a clinical point of view, oropharyngeal sensory deficits have been shown to be an important cause of dysphagia and aspiration in stroke patients. In the present study we therefore investigated effects of functional oropharyngeal disruption on the cortical control of swallowing. We employed whole-head MEG to study cortical activity during self-paced volitional swallowing with and without topical oropharyngeal anesthesia in ten healthy subjects. A simple swallowing screening-test confirmed that anesthesia caused swallowing difficulties with decreased swallowing speed and reduced volume per swallow in all subjects investigated. Data were analyzed by means of synthetic aperture magnetometry (SAM) and the group analysis of the individual SAM data was performed using a permutation test.

**Results:**

The analysis of normal swallowing revealed bilateral activation of the mid-lateral primary sensorimotor cortex. Oropharyngeal anesthesia led to a pronounced decrease of both sensory and motor activation.

**Conclusion:**

Our results suggest that a short-term decrease in oropharyngeal sensory input impedes the cortical control of swallowing. Apart from diminished sensory activity, a reduced activation of the primary motor cortex was found. These findings facilitate our understanding of the pathophysiology of dysphagia.

## Background

Human swallowing represents a complex coordinated function that is highly dependent on sensory feedback [1]. The afferent input from food or saliva is important in the initiation of swallowing [2-5]. Characteristics of the bolus such as volume or viscosity lead to a modulation of the motion sequence during deglutition. A larger bolus leads to an earlier movement of hyoid and larynx as well as an earlier opening of the upper esophageal sphincter compared to a smaller bolus [6,7]. Dysphagia, the difficulty in swallowing, can result from congenital abnormalities, structural damage, and psychiatric conditions. Neurogenic dysphagia is caused by neurologic disorders affecting central nervous, peripheral nervous or muscular structures. A sensory deficit of the pharyngeal mucosa is one of the main causes of neurogenic dysphagia in stroke patients [8,9]. Stroke related dysphagia causes aspiration and consecutive pneumonia, dehydration and malnutrition, and thereby increases mortality in these patients [8-14].

Topical anesthesia of the oropharynx causes a significant increase of swallowing duration [2-5], and a decrease of the swallowed volume and swallowing capacity (ml/s) [15] and sometimes even results in aspiration [16]. Therefore, this intervention represents an ideal model of (short-term) dysphagia due to impaired sensory feedback.

Magnetoencephalography (MEG) can monitor cortical activity with a high temporal and spatial resolution [17]. Motor tasks have been shown to result in event-related desynchronisations (ERD) of the cortical beta rhythm in cortical motor areas [18,19]. In the last few years synthetic aperture magnetometry (SAM) based on whole-head MEG has been demonstrated to be a reliable method to examine the complex function of swallowing in humans [20-22]. In the present study we employed whole-head MEG to study cortical activity during self-paced volitional swallowing with and without topical oropharyngeal anesthesia to evaluate the impact of sensory input in healthy subjects. We hypothesize a decrease of cortical beta ERD in swallowing related areas of the somatosensory system.

## Results

All participants tolerated the study. Although oropharyngeal anesthesia caused short lasting dysphagia, no coughing and especially no signs of aspiration occurred during screening tests or measurements. The oropharyngeal application of lidocaine resulted in surface anesthesia of the oral cavity and the throat. All subjects stated that sensory stimulation with a swab was not sensed in this area after application.

**Figure 1:**
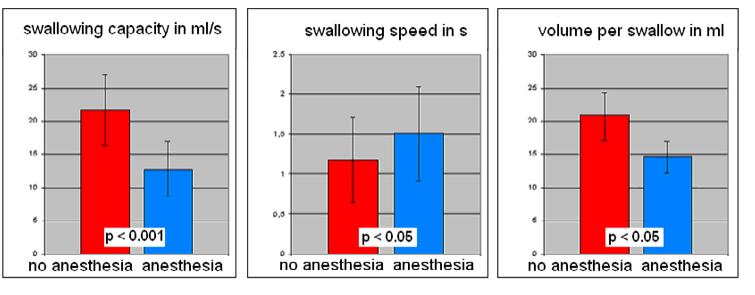
**Event-related desynchronisation**. Changes in the beta frequency band during the execution phase of a) volitional swallowing and b) pharyngeal anesthesia. The color bar represents the t-value. c) Comparison between ERD in the volitional swallowing [dark blue] and the pharyngeal anesthesia [light blue] condition.

The swallowing screening-test performed before each MEG measurement revealed signs of dysphagia after local anesthesia in all subjects. Compared to the screening-test without anesthesia, significant decrement of swallowing speed (1.18 s/swallow vs. 1.51 s/swallow; p < 0.05), reduced volume per swallow (26.2 ml vs. 18.95 ml; p < 0.05) and reduced swallowing capacity (21.66 ml/s vs. 12.78 ml/s; p < 0.001) were found [Table 1; Figure 1].

**Table 1:** Swallowing screening test

	Normal swallowing			Pharyngeal anesthesia		
	Ml per swallow	s per swallow	ml/s	ml per swallow	s per swallow	ml/s

S1	21,43	1,14	18,75	15,00	1,27	11,86
S2	15,00	0,98	15,38	12,5	1,08	11,54
S3	25,00	0,78	32,26	13,64	1,38	9,90
S4	25,00	1,01	24,71	15,00	1,10	13,62
S5	18,75	0,98	19,23	11,54	2,25	5,14
S6	16,67	0,79	21,13	21,43	1,15	18,63
S7	25,00	1,25	20,00	16,60	1,25	13,24
S8	18,75	1,12	16,75	15,00	1,59	9,46
S9	21,43	1,08	19,92	18,75	1,12	16,9
S10	75,00	2,64	28,46	50,00	2,86	17,49
Group	26,20	1,18	21,66	18,95	1,51	12,78

The swallowing screening-test was performed with and without pharyngeal anesthesia. Local application of lidocaine resulted in a significant increment of swallowing duration and a significant reduction of volume per swallow and swallowing capacity in healthy subjects.

The flow of the intraoral infusion and the amount of water swallowed during each measurement did not differ between the two conditions. Regarding the EMG data, number of swallows as well as duration per swallow did not differ between the two conditions (p > 0.05). The RMS of the EMG amplitude across the whole swallow interval (M0 - M2) showed significantly stronger EMG power in the anaesthesia condition compared to the normal swallowing condition in all ten subjects (p < 0.05) [Table 2].

**Table 2:** EMG activation

	Normal swallowing			Pharyngeal anesthesia		
	No. of swallows	Duration per swallow in s	RMS of EMG amplitude in μV	No. of swallows	Duration per swallow in s	RMS of EMG amplitude in μV

S1	93	1.86	36.98	90	2.06	53.73
S2	38	1.9	42.09	53	1.55	43.49
S3	44	1.85	68.73	28	1.43	86.11
S4	62	1.49	44.83	45	1.48	59.28
S5	53	1.54	10.40	37	1.95	20.16
S6	70	2.04	27.61	45	1.31	79.44
S7	48	1.21	131.59	38	1.48	167.48
S8	37	1.07	25.62	48	1.90	50.36
S9	58	1.68	51.34	56	1.77	162.08
S10	99	1.43	39.23	87	1.25	56.60
Group	60.2	1.70	47.84	52.7	1.62	77.87

The submental EMG was recorded during both MEG measurements. The number of swallows and the duration per swallow did not differ between the normal swallowing condition and swallowing with pharyngeal anesthesia (p > 0.05). The RMS of the amplitude over the time interval between M0 und M2 was significantly higher in the anesthesia condition compared to normal swallowing (p < 0.05).

In each individual subject, in both normal swallowing and anesthetized conditions event related desynchronisations (ERD) were found in the beta frequency band in the primary sensorimotor cortex. In the other frequency bands and other cortical areas no systematic activation was observed in either of the two conditions.

**Figure 2:**
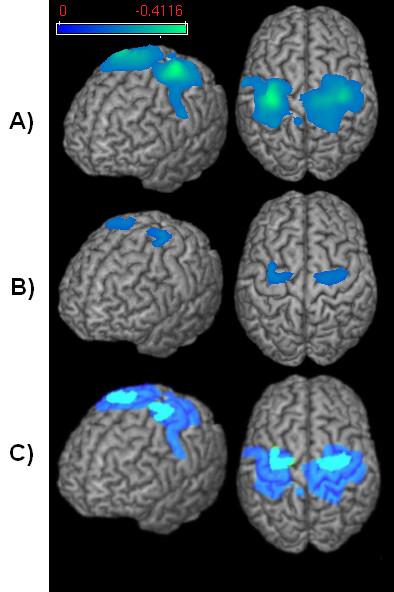
**EMG recording and resulting time phases**. Definition of active and resting stage of swallowing-related muscle activity. The EMG recording of one swallowing act is shown (surface electrodes, recording from the submental muscles). For the analysis with SAM, the beginning (M_1_) and the end (M_2_) of larynx elevation were marked. The activation phase and the corresponding resting phase were defined. To estimate the maximal null distribution a third marker (M_0_) at the beginning of preparation activity was set and two background phases were defined (Methods).

In group analysis of the single conditions, normal swallowing and anesthetized swallowing resulted in significant ERD of rhythmic brain activity in the beta frequency band. In both conditions we found significant activation (p < 0.05) in the primary sensorimotor cortex (BAs 4, 3, 1, 2) in this frequency band [Figure 2]. The maximum beta power was observed around 300 ms after the onset of swallowing related muscle activity (marker M1) in both conditions. A reduction of the sensor level beta power was observed comparing the active and control time windows in both conditions. The beta power during the resting stage did not differ between conditions (p > 0.05). The other frequency bands (alpha, high and low gamma and theta) showed no significant event-related cortical activation in the two examined conditions.

Comparison of both swallowing conditions revealed significantly less activation (p < 0.05) in the sensorimotor cortex in both hemispheres in anesthetized swallowing compared to the normal swallowing condition. The peak of the ERD was located bilaterally in the same area around the central gyrus in both conditions. The maximum pseudo-t value in the anesthesia task was 35% lower than in the swallowing task without anesthesia in the left hemisphere. In the right hemisphere the maximum pseudo-t value was 28% lower in the anesthesia task. The decrement of activation was observed mainly in the primary sensory cortex. Also the primary motor cortex was activated less in the anesthesia task compared to normal swallowing.

## Discussion

In this study we investigated the effect of functional oropharyngeal disruption on the cortical control of swallowing by means of MEG and SAM. The main finding of our study was that oropharyngeal anesthesia led to a pronounced decrease in cortical activation of the primary sensory and motor cortex as compared to volitional swallowing without anesthesia.

Cortical activation during swallowing resulted in a movement related decrease of beta power in both conditions. The decrement of activation is known as event related desynchronisation (ERD) and has been observed not only in swallowing but in several previous motor experiments [23,24]. Such movement related desynchronisations are typically observed within the range of the alpha and beta frequency band (8–30 Hz) and are somatotopically organized [25,26]. They usually occur before or during the execution of movement [27,28].

Changes of rhythmic brain activity were most pronounced in the pre- and postcentral gyri corresponding to BA 4 and BA 3, 1, 2 for normal as well as for the anesthetized swallowing conditions. These results are in line with previous studies done with MEG [20,21]; PET [29] and fMRI [30-33] showing bilateral activation of the primary sensorimotor cortex during swallowing in healthy subjects, some additionally found a left hemispheric lateralization [20,33]. The locations of the activated cortical areas observed in this study correspond to those that have been shown by TMS, fMRI, and MEG before. In a TMS study by Hamdy and coworkers, the cortical areas controlling the pharynx were located medially in the precentral gyrus [Hamdy et al., 1998, Nature Neuroscience]. An increased regional cerebral blood flow in lateral somatosensory cortex as well as in parietal areas was found in a swallowing paradigm in a PET study [29]. A swallowing task in an MEG study by Furlong and coworkers resulted in widespread beta ERD in somatosensory cortex [21].

All these studies looking at cortical processing of swallowing examined physiological deglutition in healthy subjects. In contrast our study focused on the role of sensory input and the effect of functional disruption on swallowing in human subjects. We found a significant decrement of cortical activation as well as significant increase of swallowing muscle activation after sensory input of the oropharynx had been disturbed.

The results of this study suggest that sensory input is crucial for the cortical control of swallowing execution. Thus, as mentioned above, oropharyngeal sensory deficits have been shown to cause dysphagia and aspiration in stroke patients [34,35]. Furthermore, several clinical studies confirmed the impact of sensory feedback by using oropharyngeal anesthesia [2,5,15,16]. A study using flexible endoscopic evaluation of swallowing with sensory testing (FEESST) in dysphagic patients showed that severe laryngopharyngeal sensory deficits resulted in aspiration of liquids regardless of the pharyngeal motor function [36]. This points to the outstanding role of sensory input in swallowing.

Apart from a strongly reduced sensory representation we also found a decreased motor activation in this study. A close link between sensory and motor function in human swallowing has been shown before by another FEEST study of Setzen and co-workers. They found a strong association between motor function deficits and hypopharyngeal sensory deficits in dysphagic patients [37]. In line with our results, Aviv concludes that sensory deficits lead to dysphagia by reduction of stimulus detection in the laryngopharynx and indirectly by impairing the triggering of motor actions [38]. Of special interest for our results are two further studies, one using video fluoroscopy [39] and the other fiberoptic endoscopy [40] in combination with local infiltration anesthesia. In both studies a significant increase in penetration and aspiration after administration of anesthesia was found. Based on their observations Jafari and co-workers assumed that this mainly was the result of a reduced laryngeal motor neuron drive, thereby proposing a link between impaired sensory input and disturbed motor-output. According to their suggestion, dysphagia after oropharyngeal anesthesia is not only caused by reduced sensory input directly leading to aspiration, but is also a consequence of impaired motor efferents. Interestingly, this observation is mirrored in our study, which showed apart from a reduced sensory representation also a decreased motor activation.

The second finding of this study is the significantly increased swallowing related muscle activation during anesthesia condition compared to swallowing without anesthesia. Until about 10 years ago swallowing was thought to be coordinated only by the brainstem. New functional brain imaging methods proved the influence of several cortical areas on deglutition [20,29,33]. We suppose that these findings can be explained as follows: The impairment of sensory information caused by oropharyngeal anaesthesia results in reduced cortical feedback and control, which could be shown in our study. Due to this the central pattern generators in the brainstem possibly lose an important part of cortical modulation and therefore have to take over the major part in swallowing coordination. The increased EMG-power during anaesthesia observed here might therefore reflect a less well-coordinated act of swallowing.

## Conclusion

A short-term decrease in oropharyngeal sensory input leads to diminished cortical sensory activity and also reduces activation of the primary motor cortex. This underlines the important role of sensory information on the cortical coordination of human swallowing. The increased muscle activation during anesthetized swallowing gives hint for a less coordinated control by the brainstem when cortical feedback is missing. Further studies have to show if an increased activation of distinct brainstem areas can be observed in human swallowing after oropharyngeal anesthesia.

## Methods

### Subjects

Ten healthy right-handed volunteers (7 males and 3 females, age range 22 – 60 years, mean 35.9 years) served as subjects. The local ethics committee has approved the protocol of the study. Informed consent was obtained from each subject after the nature of the study was explained in accordance to the principles of the Declaration of Helsinki.

### Topical anesthesia

Mucosal anesthesia of the oropharynx was induced by oral application of 12 puffs of 2% lidocaine spray. Subjects were asked to swallow every second spray to achieve adequate anesthesia. After this application each subject stated sufficient anesthesia of the oral cavity and faucial area. Anesthesia was additionally confirmed by the absence of touch sensation to light contact with a swab. If the soft touches were still detectable by subjects 3 additional puffs of lidocaine were applicated and the swab touches were repeated. The palatopharyngeal reflex was tested by touching the soft palate and the uvula with the swab. The aim was not to elicit pharyngeal contraction or coughing. Topical anesthesia was used twice: once before the swallowing screening test and once before the respective MEG measurement started.

### Swallowing screening test

Before MEG recording was started a dysphagia screening test was performed according to the protocol by Hughes and Wiles (1996). Each subject drank 150 ml of water from a plastic beaker. They were instructed to drink 'as quickly as is comfortably possible'. Subjects were observed from the side, and the number of swallows counted by observing the movements of the thyroid cartilage. A stopwatch was started when the water first touched the bottom lip, and stopped when the larynx came to rest for the last time [41]. The swallowing screening test was performed with and without topical anesthesia applied as described above.

### Intra-oral infusion

To facilitate volitional swallowing during MEG recording water was infused into the oral cavity via a flexible plastic tube 4.7 mm in diameter attached to a fluid reservoir. The reservoir bag was positioned about 1 m above the mouth of each subject when seated. The tip of the tube was placed in the corner of the mouth between the buccal part of the teeth and the cheek. The tube was gently fixed to the skin with tape. The side chosen for tube placement was alternated between subjects. The infusion flow was individually adjusted to the subject's request and ranged between 8 and 12 ml/min. The aim was to establish a swallowing frequency of four to six times per minute.

### MEG recording

During 15 min of MEG recording the subject swallowed self-paced without external cue. Swallowing acts were recorded and identified by electromyographic recording. The MEG recording was done with and without topical anesthesia in all 10 subjects investigated. In 5 subjects the normal swallowing condition was done first, the other five subjects started with topical anesthesia. In these cases we waited about 1 hour after anesthesia had been performed and ensured that the swallowing screening test had normalized before we started with the normal swallowing condition.

MEG data were collected using a whole head 275-channel SQUID sensor array (Omega 275, CTF Systems Inc.) installed within a magnetically shielded room. Magnetic fields were recorded with a sample frequency of 600 Hz. The data were filtered during acquisition using a 150 Hz low-pass filter. Recordings were performed while subjects were seated in a comfortable upright position and watching a self-selected silent movie.

### EMG recording

Surface EMG was measured with two pairs of bipolar skin electrodes (Ag-Ag-Cl) placed on the submental muscle groups [42,43]. The electrodes were connected to a bipolar amplifier (DSQ 2017E EOG/EMG system, CTF Systems Inc., Canada), and the nominal gain was set at 1. EMG data was high pass filtered with 0.1 Hz before markers were manually set.

### Anatomical MRI

MRI data were acquired on a 3.0 T Scanner (Gyroscan Intera, Philips Medical Systems, Best, The Netherlands) with a standard head coil. T1-weighted sagittal anatomical images with in-plane resolution of 512 × 512 (0.6 × 0.6 mm resolution) and 320 slices (0.5 mm thickness) were recorded using spoiled gradient echo imaging.

### Data analysis

**Figure 3:**
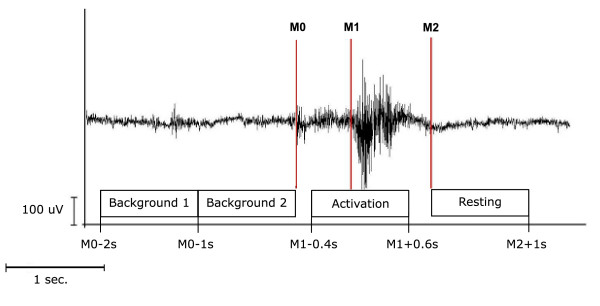
**Swallowing screening test**. Comparison between the two conditions (swallowing with and without topical anesthesia) in the swallowing screening-test. The screening-test reveals a significant decrease in swallowing capacity and volume per swallow and increase in swallowing speed after anesthesia was performed.

According to the individual EMG signal the beginning of laryngeal elevation (M_1_) and the end of the task-specific muscle activity (M_2_) were marked for every single swallow in each subject. The beginning of laryngeal elevation was defined as an increase of greater than 200% in amplitude or frequency of the EMG signal after an initial increase of EMG activity defining the beginning of the preparation phase. The end of task-specific muscle activity was defined as greater than 50% decrease in amplitude or frequency of the EMG signal. To estimate the maximal null distribution (see below) a third marker was set in order to distinguish background activity from the beginning of the preparation phase (M_0_) [Figure 3]. In all subjects, the movement stage lasted longer than 1 s in more than 90% of the trials. Only these trials were taken into account. In contrast to a former study by our group [20], where 1.5 sec. per swallow was taken into account, here the swallowing duration was shorter and more variable in the subjects investigated. For further analysis time intervals were defined as following:

(1) Movement stage: -0.4 to 0.6 s in reference to M_1_

(2) Resting stage: 0 to 1 s in reference to M_2_

(3) Background stage 1: -2 to -1 s in reference to M_0_

(4) Background stage 2: -1 to 0 s in reference to M_0_

Four percent of the trials were rejected due to overlap between (1) and (2) or between (4) and (2) of the subsequent swallow.

Synthetic aperture magnetometry (SAM), a minimum-variance beamformer, with an integrated step for the estimation of dipole orientation was used to analyze the recorded MEG data [44]. In contrast to other MEG source localization methods beamforming does not rely on averaging and therefore allows the analysis of evoked and induced brain activity. Like fMRI SAM calculates volumetric maps of brain activation and allows the application of similar paradigms as used in fMRI investigations. But in contrast to fMRI, SAM can benefit from the millisecond resolution of MEG. While fMRI monitors changes [29]in blood flow with the BOLD effect MEG directly measures neuronal activity.

SAM has proved to be a valid method leading to reliable results in several sensorimotor [45,46] as well as swallowing studies [20,21].

In this study the recorded MEG data were filtered within five different frequency bands: theta (4–8 Hz), alpha (8–13 Hz), beta (13–30 Hz), low gamma, (30–60 Hz), high gamma (60–80 Hz).

SAM was used to generate a 20 × 20 × 14 cm volumetric pseudo-t images [47] from the filtered MEG signals, with 3-mm voxel resolution. A pseudo-t value cancels the common-mode brain activity by subtracting the source power found in a defined control stage from the source power in the active stage. To account for uncorrelated sensor noise, this difference is normalized by the mapped noise power [47,48]. For analyzing cortical activity during the movement stage (1) the corresponding resting stage (2) served as control.

Group analysis of multiple subjects' data was performed as previously published [45,46,49,50]. Briefly, the individual MRIs were first transformed into a common anatomical space using SPM2. Then the spatial normalized activation maps were obtained by applying this transformation to the individual SAM volumes.

For analysis of single conditions the significance of activated brain regions was investigated by the permutation test method described by Chau and co-workers (2004). The maximal null distribution was estimated here by comparing background stage 1 (active) and 2 (control) [50,51]. For comparison of both conditions a standard permutation test for paired samples was performed [51]. The sensor level power in frequency bands with significant differences between the conditions were further analyzed using the Fast Fourier Transform method to exclude systematic errors by differences between the control stages.

## Authors' contributions

IT has made analysis and interpretation of data and drafted the manuscript, OS has made analysis and interpretation of data and was involved in drafting the manuscript, KS and SS have made contributions to conception and design and did data acquisition, AW has made substantial contributions to conception and design, CP revised the manuscript critically for important intellectual content, RD made substantial contributions to conception and design, and has given final approval of the version to be published.

IT is funded by the Deutsche Forschungsgemeinschaft.
